# Structure and Bottom-up Formation Mechanism of Multisheet Silica-Based Nanoparticles Formed in an Epoxy Matrix through an *In Situ* Process

**DOI:** 10.1021/acs.langmuir.1c01363

**Published:** 2021-07-18

**Authors:** Francesco Branda, Aurelio Bifulco, Dieter Jehnichen, Dambarudhar Parida, Robin Pauer, Jessica Passaro, Sabyasachi Gaan, Doris Pospiech, Massimo Durante

**Affiliations:** † Department of Chemical, Materials and Production Engineering (DICMaPI), University of Naples Federico II, Naples 80125, Italy; ‡ Department Nanostructured Materials, Leibniz-Institut für Polymerforschung Dresden e. V., Hohe Str. 6, Dresden 01069, Germany; § Laboratory for Advanced Fibers, Empa Swiss Federal Laboratories for Materials Science and Technology, Lerchenfeldstrasse 5, St., Gallen 9014, Switzerland; ∥ Advanced Materials and Surfaces, Empa, Swiss Federal Laboratories for Materials Science and Technology, Dubendorf CH-8600, Switzerland; ⊥ Department Polymer Structures, Leibniz-Institut für Polymerforschung Dresden e. V., Hohe Str. 6, Dresden 01069, Germany

## Abstract

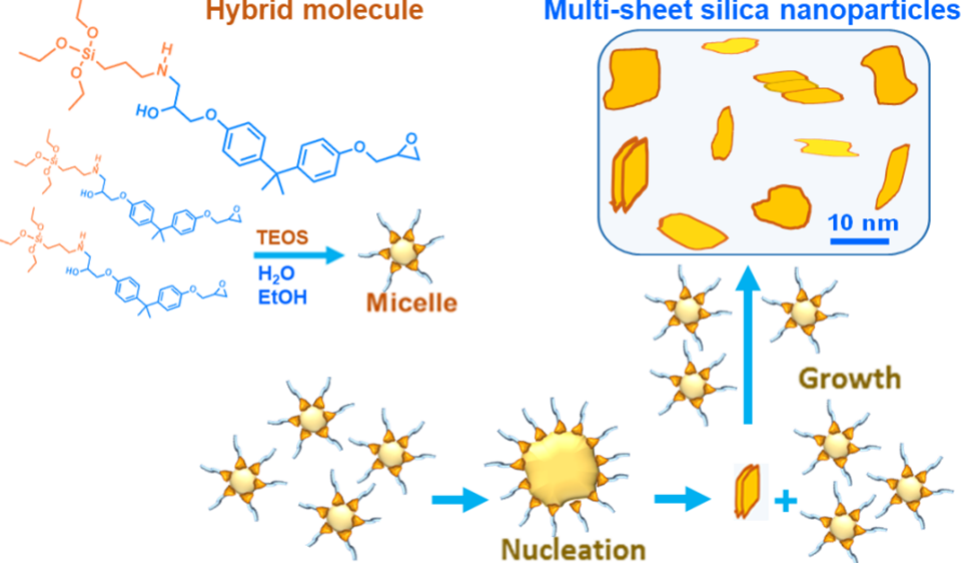

Organic/inorganic hybrid composite materials with the dispersed phases in sizes down to a few tens of nanometers raised very great interest. In this paper, it is shown that silica/epoxy nanocomposites with a silica content of 6 wt % may be obtained with an “*in situ*” sol–gel procedure starting from two precursors: tetraethyl orthosilicate (TEOS) and 3-aminopropyl-triethoxysilane (APTES). APTES also played the role of a coupling agent. The use of advanced techniques (bright-field high-resolution transmission electron microscopy, HRTEM, and combined small- and wide-angle X-ray scattering (SAXS/WAXS) performed by means of a multirange device Ganesha 300 XL+) allowed us to evidence a multisheet structure of the nanoparticles instead of the gel one typically obtained through a sol–gel route. A mechanism combining in a new manner well-assessed knowledge regarding sol–gel chemistry, emulsion formation, and Ostwald ripening allowed us to give an explanation for the formation of the observed lamellar nanoparticles.

## Introduction

Recently, very great interest has been devoted to the development of organic/inorganic hybrid (HOI) composite materials with the dispersed phases in these materials in sizes down to a few tens of nanometers.^1−12^ Such materials find widespread applications spanning from the field of protective coatings to electrochemistry, catalysis, biomaterials, energy storage devices, etc.^2,3,13,14^ Many applications take advantage of the “nano” size, i.e., an enhanced catalytic activity as the result of the specific surface increase. Very often, unprecedented materials were produced whose properties are not simply a combination of the two components alone.^15^ A common way to develop these materials is the use of the sol–gel synthesis route.^16−19^ The mild synthesis conditions in the sol–gel process allow generating an inorganic phase in the presence of the monomer or the polymer through the so-called “*in situ*” process, in which both phases are formed successively in one procedure.^3,20^ This is of great interest and was recently actively explored also in the case of non silicatic fillers.^21,22^ When dealing with sol–gel chemistry, if the reaction rates are comparable, an interpenetrating network may also be obtained from the simultaneous reactions of the organic monomer and the sol–gel precursor.^20^ In addition, the sol–gel route offers the formation of an inorganic phase starting from simple low-molecular-weight precursors, allowing a nanolevel molecular design of the final structure. This “bottom-up” approach is necessary for HOI production.^2,15^ It is also worth mentioning here that the HOI development took advantage of the availability of instruments for nanoscale observation and characterization, in particular high-resolution transmission electron microscopy (HRTEM) and small-angle X-ray scattering (SAXS). One of the most studied HOI systems is based on epoxy/silica, which is characterized by silica nanoparticles well dispersed in a cured epoxy matrix.^4−6^ Recently, the authors exploited the sol–gel chemistry to produce a silica/epoxy HOI with an inorganic content up to 6 wt %.^23−26^ Two silica precursors were used: tetraethyl orthosilicate (TEOS) and (3-aminopropyl)-triethoxysilane (APTES). APTES also played the role of a coupling agent between silica and the epoxy matrix by allowing chemical reactions between the components. The molar ratio TEOS/APTES was kept constant (2.3:1) in these studies. The experimental results (transmission electron microscopy, TEM; small-angle X-ray scattering, SAXS; Fourier-transform infrared spectroscopy, FTIR; nuclear magnetic resonance, NMR; and dynamic mechanical analysis, DMA) proved that the dispersed phase consisted of silica particles, a few nanometers in size, well dispersed in the epoxy network. Despite the very low silica content, the HOI exhibited an excellent fire behavior: absence of dripping during a vertical flame spread test, formation of a continuous and stable char, and a reduction in the heat release rate (HRR), down to 60% of the neat epoxy resin one.^23−26^

In this work, it is shown that the procedure employed, which followed previous works,^23−26^ allows us to prepare silica/epoxy composites with a silica content of 6 wt % and TEOS/APTES molar ratio as low as 1.25. The structure of the silica nanoparticles formed is studied with the aid of bright-field HRTEM as well as X-ray scattering over an unprecedented *q* range (0.02–25 nm^–1^, see the Experimental Section). These are the most suitable techniques to have detailed information on the structure of nanocomposites.^27^ The sol–gel route performed in the temperature range until 80 °C normally results in the formation of silica gel, in particular gel particles in an alkaline environment (Stöber method).^28−33^ The hydrolysis and polycondensation reactions in the case of the very popular alkoxide precursors Si(OR)_4_ can be written as^28−33^
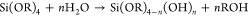
1
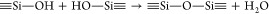
2
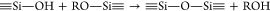
3When a sufficient number of siloxane (−Si–O–Si−) bonds are formed, further silicatic chain growing is prevented and a gel forms. On the contrary, in the present study, silica based nanoparticles with a well-defined sheet structure are obtained. A mechanism of formation of the observed multisheet silica-based nanoparicles is also proposed.

## Experimental Section

### Materials

Tetraethyl orthosilicate (TEOS, >99%), (3-aminopropyl)-triethoxysilane (APTES, >98%), and ethanol (ACS reagent, anhydrous) were purchased from Sigma-Aldrich (Switzerland). A two-component epoxy resin system (SX10 by MATES S.r.l., Milan, Italy), consisting of bisphenol A diglycidyl ether (DGEBA) and modified cycloaliphatic polyamines, was used for fabricating composite laminates.

### Synthesis of Epoxy/Silica Nanocomposite

APTES and TEOS were used as silica precursors and added to the commercial two-component epoxy resin system by following the same synthesis route reported in the literature.^24,26^ Thus, an “*in situ*” sol–gel synthesis was promoted prior to the addition of the epoxy hardener. The TEOS/APTES weight ratio was kept equal to 1.25. So as indicated in the second step reported below, silica formation required a higher temperature (80 °C instead of room temperature) and reflux conditions.^34^ The synthesis was performed in one pot involving the following three steps:

- A mixture of epoxy (DGEBA) and APTES with a weight ratio of epoxy/APTES of 100/5 was stirred vigorously at 80 °C for 2 h to get a silanized epoxy.
- TEOS, distilled water, and ethanol (EtOH) were added to the silanized epoxy and subsequently stirred vigorously at 80 °C under reflux for 90 min, and the reaction vessel was then opened and kept at 80 °C for 30 min in order to remove ethanol and water.
- The amount of hardener needed for the curing was then added to the mixture at room temperature and mixed for 5 min.
- The resulting mixture was degassed under vacuum and poured into a Teflon mold. The curing process was carried out at 30 °C for 24 h; then, the sample was post-cured at 80 °C for 4 h.

The silica content estimated from the stoichiometry was 6 wt %, and the hybrid sample was coded as **EPO6Si_1.25** (“EPO” is the acronym for the epoxy resin system, “6Si” the silica content, and “1.25” represents the TEOS/APTES weight ratio) throughout the paper.

### Characterization and Investigation Techniques

#### Fourier Transform Infrared Spectroscopy

FTIR transmittance spectra were recorded with a Nicolet 5700 FTIR spectrometer (Thermo Fisher, Waltham, MA, USA), using a single-reflection attenuated total reflectance (ATR) accessory with a resolution of 4 cm^–1^ and 32 scans and a Thermo Scientific OMNIC Software Suite (v7.2, Thermo Fisher, Waltham, MA, USA, 2005). All the obtained spectra were normalized to the strong absorption bands at 1607 and 1509 cm^–1^, related to the bonds of the benzene rings present in the epoxy resin structure, that are not expected to change after the curing reaction.

#### Dynamic Mechanical Analysis

DMA tests were carried out on a DMA3300 (TA Instruments). The tests were run in a three-point bending mode with a span of 40 mm and a frequency of 1 Hz; the width of samples was about 10 mm and the temperature was ramped from 25 to 100 °C at a heating rate of 3 K/min. The analysis was repeated on three samples of each composition.

#### Transmission Electron Microscopy

TEM images of composite samples were recorded using a TEM/STEM JEOL JEM 2200 fs microscope operating at 200 kV. Prior to TEM analysis, powders of the sample were prepared and dispersed in water and a drop of finely dispersed sample was put on a Lacey Carbon film copper TEM grid. The TEM grid with the sample droplet was dried overnight in an oven at 40 °C. 50 particles at random locations were analyzed by Image J to determine the particle size and distribution. HRTEM images were used to determine the lattice plane distance using Image J software.

#### Combined Small- and Wide-Angle X-ray Scattering

SAXS/WAXS experiments were executed by means of the multirange device Ganesha 300 XL+ (SAXSLAB ApS, Denmark/USA). We used Cu Kα radiation (μ-focus tube 50 kV, 600 μA; monochromatization with bifocal Göbel mirror). Scattering intensities were accumulated by a 2D-detector Pilatus 300 K (pixel size 172 × 172 μm^2^). The path of rays, sample, and detector are completely under vacuum (*p* < 5 × 10^–2^ mbar, thus no background scattering by air). For the actual investigations, a 2-slit configuration and a beamstop with a 2 mm diameter was applied.

The experiments were realized in asymmetric transmission. The bulk sample was mounted free-standing, whereas the powder sample was enwrapped by thin aluminum foil. The primary data (intensity I) were recorded as 2D-scattering frames *I*(*qx*, *qy*) in three scattering ranges (with overlap), which were distinguished by different distances sample-detector. The intensities were corrected by sample absorption. Bragg’s law is the basic eq 4 for scattering experiments:
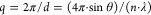
4where *d* is the lattice plane distance, θ is the half scattering angle, *n* is the order of reflection, and λ is the wavelength (Cu Kα radiation with 0.1542 nm). The total radial intensity profile *I*(*q*) was calculated via azimuthal averaging of the 2D frames and subsequent by merging of the partial intensity profiles; q is the (radial) scattering vector. For samples (especially polymeric samples) having isotropic scattering behavior in three-dimensional space (no any orientation effects), the measured intensity data near-zero diffraction angle (SAXS region) have to be corrected in general by a factor containing a sin(2θ) element or its squared form sin^2^(2θ) to reduce the intensities at small angles, which were generated from periodic structures (Gaussian or Lorentzian peaks with a peak maxima in correlation to the Bragg periodicity) as well as dispersed particle systems (Gaussian peaks around zero point). Applying Lorentz correction is helpful to enhance the visibility of the structures with periodicities (e.g., ordered phase separation), but it degrades the feature of particle scattering.^35−37^ Total radial intensity profiles were presented after Lorentz correction using the simplified formula 5:
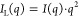
5

as lg *I*_L_(lg *q*) and/or lg *I*_L_(lg *d*).

## Results and Discussion

**Figure 1:**
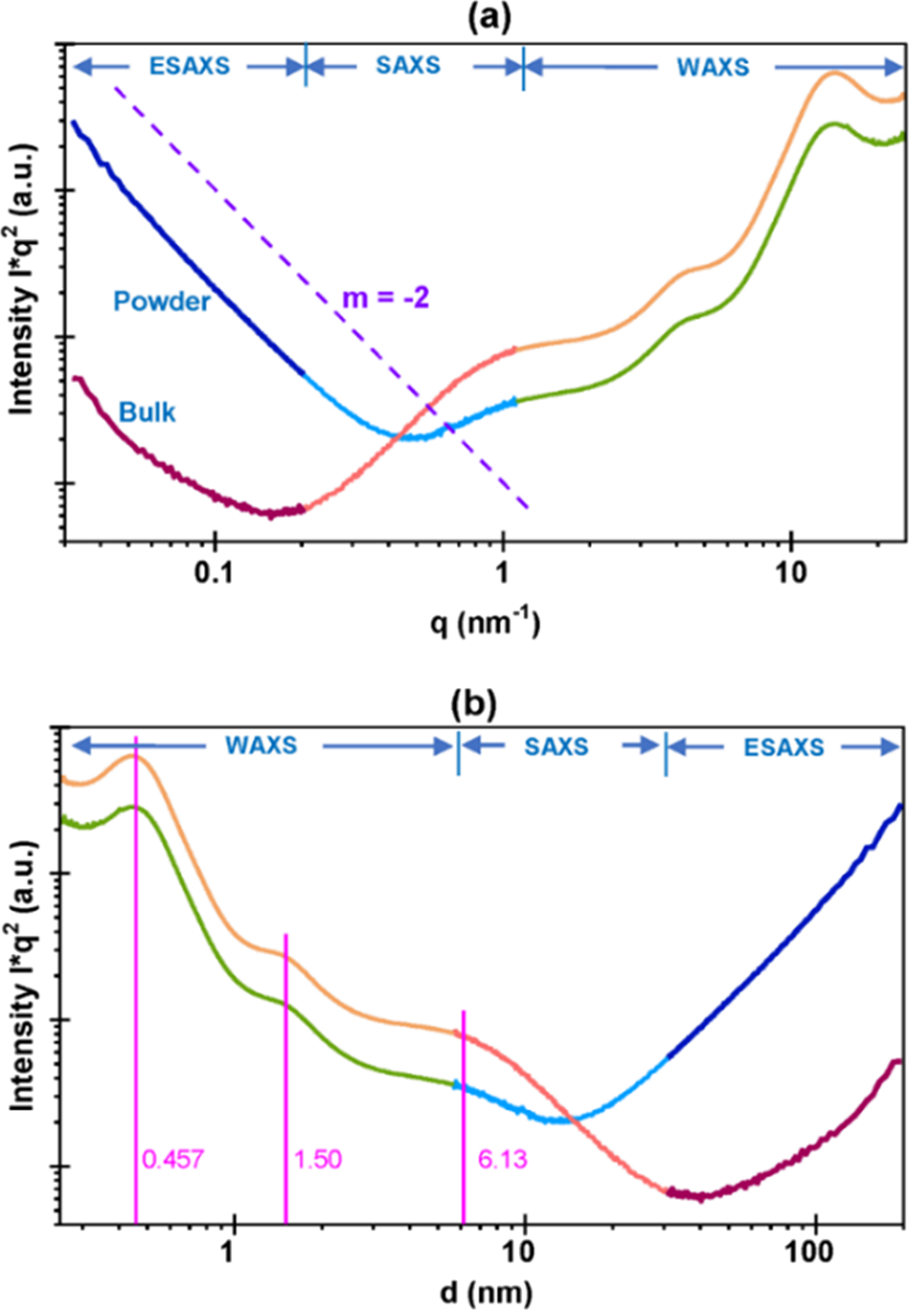
SAXS/WAXS curves: (a) intensity vs *q* and (b) intensity vs *d* of epoxy/silica nanocomposites (bulk and powder curves). Both figures show radial intensity profiles after Lorentz correction. This correction was applied to enhance the visibility of the structures with periodicities (e.g., ordered phase separation) as well as dispersed particle systems. In Figure 2a–c, the HRTEM micrographs are displayed.

In Figure S1, the FTIR spectra of epoxy (**EPO**) and its composite (**EPO6Si_1.25**) are reported together with the uncured resin (**EPO_uncured**) for comparison. The band at 914 cm^–1^ present in the FTIR of uncured resin, which is attributed to the epoxy ring, almost disappears in the cured samples, implying the almost complete epoxy cure.^34,38^ Moreover, the increase, although small, of the band at 1070 cm^–1^ in the composite spectrum agrees with the presence of silica. The composite sample (in both powder and bulk form) was examined by X-ray scattering in the WAXS, SAXS, and extremely small-angle region. The results are reported in Figure 1a,b and Figure S2 (linear scattering curves: *I* = *I*(2θ)). The following observations can be summarized:

- WAXS shows only amorphous scattering with one broad scattering maximum at ∼18° in 2θ and one weak scattering maximum at ∼43°-44° caused by the epoxy matrix.
- SAXS shows two further maxima (∼5.8° and ∼1.25°), that could be caused by unknown phase separation between the epoxy matrix and dispersed nanoparticles and/or the amorphous scattering from a special compound (organic/inorganic hybrid).
- ESAXS at very small scattering angles shows the typical pattern of well-distributed nanoparticles. This particle scattering is caused by the silica particles in the bulk and silica alone (in powder, in vacuum). Thus, the intensity of particle scattering in the powder sample must be quite high compared to the bulk. The slope – 2 respective 2 in the log–log curves (Figure 1a,b) is the result of “strong” phase boundaries caused by the differences in phase contrast.

**Figure 2:**
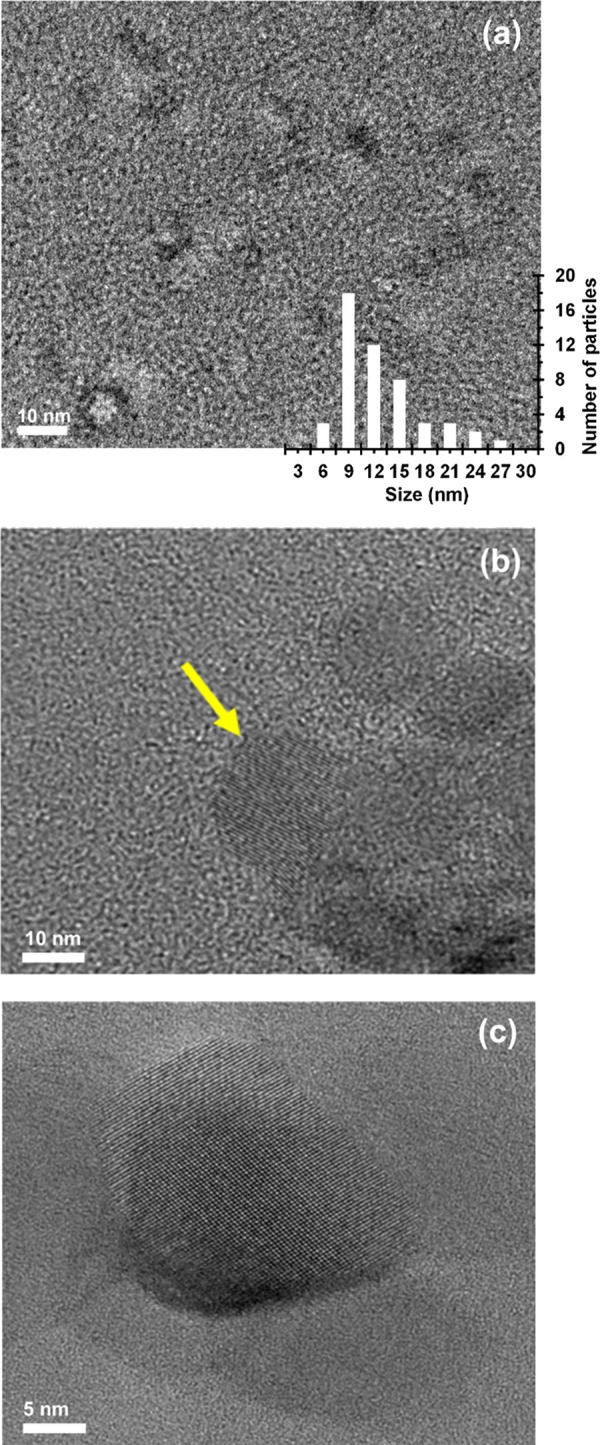
HRTEM images of silica/epoxy nanocomposite. (a) nanoparticles with their size distribution; (b, c) nanoparticles at higher magnification.

Figure 2a–c, the HRTEM micrographs are displayed. Figure 2a shows very tiny nanoparticles of different sizes immersed in an amorphous matrix. In addition, Figure 2a shows the nanoparticle size distribution determined by measuring 50 particles at different locations; the size spanning is from 6 to 27 nm. The particle sizes agree quite well with the size values obtained from SAXS investigation, 5–9 nm (see Figure 1b). Figure 2b and Figure S3 show that, at higher magnification, the nanoparticles appear to be consisting of equally spaced sheets, similar to the nanocrystalline structures observed through HRTEM by other researchers.^39^ The sheet thickness determined by a method described in the literature^39^ shown in Figure S3 is 0.34 nm, which is quite close to the lower value given by SAXS investigation (i.e., 0.485 nm, Figure 1b). Some particles have a different aspect (Figure 2c). It is worth pointing out that when exerting the mechanical action to prepare the sample, the fracture surface should propagate in the polymeric matrix or at the polymer/silica interface; therefore, no deformation of silica-based nanoparticles should occur. As a consequence, the aspect modifications should not be related to the preparation of samples. Otherwise, the SAXS analysis is in very good agreement with the TEM observations about nanoparticle size distribution, also suggesting no artifacts arising from TEM sample preparation. An explanation for the different aspect ratio of particles in Figure 2b,c is reported in the following. It appears as if something having a slightly different texture is deposited on the particle surface shown in Figure 2c. At the border of the two phases, the superimposed one progressively changes in the well-defined sheet structure observed in Figure 2b. This appears to suggest that the formation of the nanoparticles may occur through the well-known nucleation/growth mechanism proposed initially for inorganic glass crystallization,^40−44^ which was also applied to crystallization of macromolecules.^44^ In particular, the multisheet nanoparticles’ growth would occur through addition of “smaller structural units” present in the matrix to the already formed crystal surface. The presence of multisheet nanoparticles of different sizes (see Figure 2a) would also be in agreement with a mechanism of nucleation and crystal growth occurring simultaneously: earlier nuclei would have more time to grow with respect to late nuclei.

**Scheme 1:**
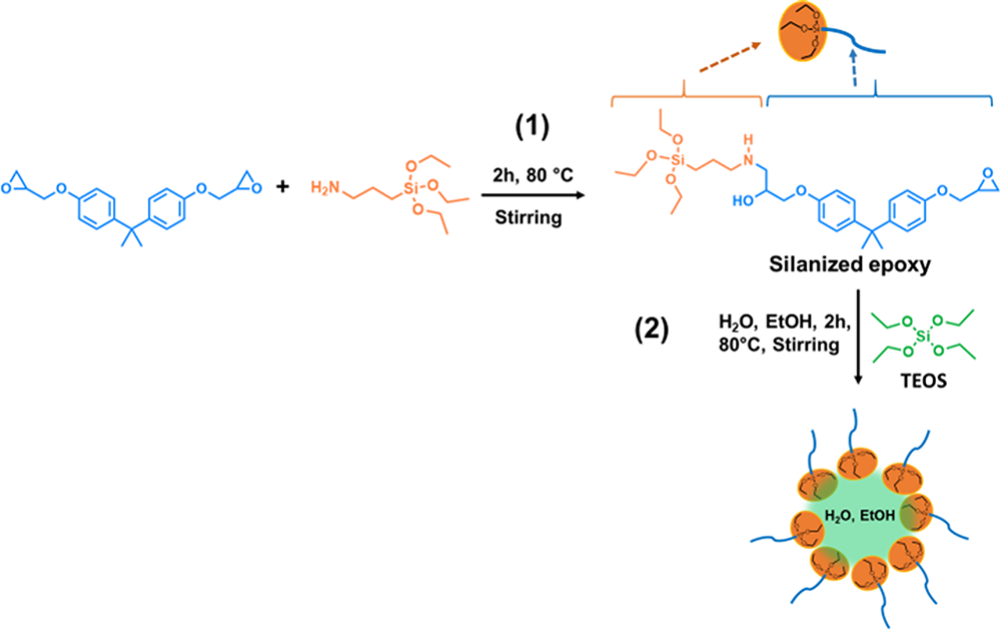
Formation Mechanism of the Silanized Epoxy Hybrid in Step 1 and Proposed Formation Mechanism of Micelle-like Nanodroplets in Step 2

A simple hypothesis about the nature of the “smaller structural units“ on the basis of the growth process and a more detailed mechanism of the formation of nanoparticles of Figure 2a is proposed in the following, where the terms nanomaterial, aggregate, and agglomerate will be used according to the “EU recommendation 696/201″ (ISO 26824, ISO TS 27687):^39,45^ nanomaterial is “a minute piece of matter with defined physical boundaries”; an aggregate is “a particle comprising of strongly bound and fused particles”; an agglomerate is “a collection of weakly bound particles or aggregates where the resulting external surface area is similar to the sum of the surface areas of the individual components”. At first, it is worth reminding that, according to the above described procedure, during the first synthesis step, APTES is left to react with the DGEBA resin at 80 °C for 2 h in a molar ratio of DGEBA/APTES of 0.294 mol of DGEBA to 0.0226 mol of APTES (see the Experimental Section), yielding a molar ratio of epoxy/NH_2_ of 26. That means some hybrid molecules (i.e., silanized epoxy species) are expected to be formed through reaction of the APTES amino group and DGEBA oxirane ring (Scheme 1, step 1). Due to the low concentration of NH_2_ groups, the formation of mostly monofunctionalized DGEBA molecules is expected, as shown in Scheme 1. The occurrence of this reaction (in the same experimental conditions of the present paper) was well proven through FTIR.^34,46^ These hybrid molecules have statistically at one end three ethoxy groups that can undergo hydrolysis reactions and result in higher hydrophilicity. At the other end, they bear one DGEBA molecule that, in contrast, makes the molecule “epoxyphilic”. In the second step, TEOS, water, and ethanol were added. Considering their structure, the hybrid molecules might play a role similar to that of amphiphilic molecules in water/oil systems. Micelle-like nanodroplets would form (see Scheme 1, step 2) and an epoxy-based nanoemulsion would form so as already hypothesized for the POSS-bisDOPO/epoxy system thanks to “surfactant-like” POSS-bisDOPO molecules.^6^

In particular, they might stabilize very tiny water/alcohol nanodroplets inside the epoxy resin that would play the role of the continuous phase of the emulsion. In these droplets, the typical sol–gel hydrolysis and polycondensation reactions might take place with the formation of very tiny silica particles (see Scheme 1, step 2).^20,47^ Considering that, during the second synthesis step, the system is characterized by great fluidity, the micelle-like nanodroplets, once formed, may be expected to agglomerate and aggregate to the surface of the multisheet silica-based nanoparticles and form the deposited phase seen in Figure 2c. These aggregates might also be continuously converted in the well-defined nanosheet structure of Figure 2b through a solution-mediated transport mechanism and Ostwald ripening process discovered at the beginning of the 20th century. According to IUPAC, this process is due to “dissolution of small crystals or sol particles and the redeposition of the dissolved species on the surfaces of larger crystals or sol particles”.^48^ This process is recognized to preside over the synthesis of complex nanomaterials.^49^ A tentative hypothesis is that, as long as the nanodroplets deposited on the multisheet nanoparticles surface keep the solvent inside, the Ostwald ripening process allows them to keep on growing with the well-defined sheet structure shown in Figure 2b. The described agglomeration, aggregation, and Ostwald ripening mechanism processes might also be the basis of the nucleation of the multisheet nanoparticles: a “micelle-like nano-droplet” of appropriate size might well generate the nuclei of the multisheet phase. When, in the third step, the curing agent is added, the structure is “frozen” without the possibility of further change. The hybrid composite materials were subjected to dynamic mechanical analysis (DMA). The analysis was repeated on three samples obtaining, for the same composition, very similar curves. In Figures S4 and S5, the representative tan δ and storage modulus *E*’ plots against temperature of neat epoxy and hybrid materials (i.e., **EPO6Si_1.25**) are reported. The hybrid shows an increase of both glass transition temperature (from 80 ± 1 to 84 ± 1 °C) and *E*’ modulus mean values (from 2.7 ± 0.2 to 3.2 ± 0.2 · 10^9^ Pa) corresponding to an increase of 19%. The increases may be ascribed to the strong interaction between the silica nanoparticles and epoxy matrix through the hybrid molecules formed in the first step and located at the epoxy/silica interface. As it is known,^13,18,20,50,51^ the hybrids are classified on the basis of the type of bonds at the O/I interface as “class I” in the case of weak bonding and “class II” in the case of strong bonding. The DMA results suggest classifying the present hybrid as “class II”.

In the following, a more detailed hypothesis concerning formation of the nuclei is developed. First of all the sheet structure of the “multi-sheet silica based nanoparticles “suggests that the hybrid APTS/epoxy molecule takes part to their formation. In fact, if formed only from TEOS, silica particles would be expected to form. Therefore, the nanoparticles are expected to have a hybrid organic inorganic (O/I) nature. It is also worth pointing out that, as a consequence, the nanoparticles may also be expected to possess a high affinity for the epoxy matrix (we could say they are “epoxophilic”) owing to the presence, also at the surface, of the hybrid APTS/epoxy molecules, exposing epoxy groups externally. This is, otherwise, in good agreement with the observed and above discussed change of glass transformation temperature. We must expect that, owing to its nonpolar nature, TEOS dissolves in epoxy (where water cannot) and hydrolyzes when entering the micelles. Of course, also the silane head of the hybrid APTS/epoxy molecule constituting the micelle should be fully hydrolyzed. If aggregation of micelles is admitted, a simple explanation is found to the involvement of both TEOS compared to the hybrid APTS/epoxy molecule in the formation of nuclei. It is useful reminding, in fact, that the specific surface of a droplet decreases when its radius increases. Therefore, the required number of hybrid molecules at the droplet border is expected to decrease when the micelles do aggregate. The hybrid molecules subtracted to the surface are available for the nuclei formation. Finally, the formation of the “epoxophilic” hybrid O/I nanoparticles may well cause separation of the micelle aggregate into a nanoparticle (the nuclei of the new phase) and one or more new micelles containing the water residual from the hydrolysis and condensation reactions of alkoxy groups (of TEOS or APTS).

A rough estimate proves that the number of hybrid APTS/epoxy molecules available when micelle aggregation does occur is significant. It is well known that the specific surface of a sphere is S/V = 3/R or S/m = 3/ρR (where S, V, R, m, and ρ are the surface, volume radius, mass and density, respectively). As a consequence, if, upon aggregation, the micelle diameter doubles, the specific surface of the “aggregated micelle” would be 2 times lower than the micelle one. This means that the mass of solution contained in them would occupy a volume having a surface 2 times lower. So also, should decrease the need of hybrid APTS/epoxy molecules present at the micelle surface. Therefore, a significant percentage (50%) of the hybrid APTS/epoxy molecules initially involved in the formation of micelle would be available for the nuclei formation.

The nanoparticles growth would occur when micelles aggregate with nanoparticles. In this case, at least a part of the hybrid APTS/epoxy molecules may be involved with the nanoparticles growth. It is worth pointing out that SAXS results well support the mechanism. In fact SAXS shows two maxima. Considering the related d-values, the first one (at ∼1.25°) could be caused by a phase separation between epoxy matrix and dispersed nanoparticles (around 6 nm in size) so as already discussed. The second one (at ∼5.8°) could well be evoked by amorphous scattering from a special compound (organic/inorganic hybrid) predicting for the residual micelles a diameter of about 1.50 nm. The mechanism allows to give explanation also to NMR results reported, recently, for a composite of very close composition, prepared with exactly the same procedure.^26^ It was found: (a) the ^29^Si NMR spectra of studied hybrid material showed that most of silicon atoms appeared to have been fully involved in the condensation reactions; (b) the ^13^C CPMAS NMR spectra showed that the intense ethoxy peaks were not detected in the most shielded alkyl-C region, suggesting an approximately complete hydrolysis of ethoxy groups in both APTES and TEOS reagents. This is quite surprising taking into account^30,31^ that usually a significant residual organic fraction does remain from the synthesis owing to incomplete hydrolysis and polycondensation reaction (see above reported reactions (1) and (3)). As long as the proposed mechanism is valid a simple explanation may now be given. It is hypothesized, in fact, (see last 3 lines of column 1 of page 4) that TEOS dissolves in epoxy (where water cannot) and hydrolyses when entering the micelles where a high H_2_O/TEOS ratio is, therefore, expected. As a consequence, complete hydrolysis is awaited. The same, of course, is forecast for the silane head of the hybrid APTS/epoxy molecule. The micelle would form, in fact, just thanks to the strongly polar character of the hydrolyzed hybrid molecule.

## Conclusions

Finally, HRTEM and SAXS allow us to have an insight into the nanoparticles (few nanometers in size), evidencing a multisheet structure that would form as the result of agglomeration and aggregation of micelle-like nanodroplets generated thanks to the hybrid molecules resulting from the reaction of epoxy with the coupling agent (i.e., APTES). The micelles would assure the proper environment (presence of water which is not soluble in the resin) where the “small structural units” (required by Tammann theory for the formation of nuclei and crystal growth) may form. Nuclei would form on aggregation of micelles; crystals would grow thanks to aggregation with micelles. The mechanism may explain the experimental results, in particular formation of particles with a multisheet instead of a gel structure. It is, otherwise, worth mentioning that the relevant effects of the presence of “surfactants” on the sol–gel chemistry were recently recognized: new mesoporous gel textures were obtained when sol–gel network architectures were controlled with the aid of the polymeric chain surfactant molecules.^14,20,52,53^ However, in the new hypothesized mechanism, the “solvent” and “surfactant” are not the common ones but, respectively, an epoxy resin at relatively high temperature (80 °C) and hybrid molecule obtained through the reaction of the “coupling agent″ with the epoxy monomer. In the present case, silica-based nanoparticles are formed “*in situ*” in DGEBA starting from simple precursor (TEOS and APTS) molecules both of which, to be hydrolyzed, need water that is not soluble in DGEBA. The proposed mechanism allows to overcome the problem through the formation of the micelles making available the proper environment where the “small structural units” (required for the formation of nuclei of the silica based nanoparticles and their growth) may form. It is worth reminding that something similar was hypothesized^54^ to occur in the mechanism of formation of mesoporous silica nanoparticles with radial wrinkle structure, which are typically formed in ternary systems of polar solvents (represented by water), nonpolar solvents (oil), and surfactants. The reaction mixtures in those cases correspond^54^ to the Winsor III system. It was hypothesized^54^ that TEOS dissolved in the oil layer comes into contact with the water at the emulsion interface where hydrolysis and condensation reactions occur. The mechanism allows us to explain, also, the peculiarities of recently published NMR results. Finally, the mechanism involves concepts borrowed from the nucleation/growth mechanism proposed initially by Tammann and the Ostwald ripening process.

## Supporting Information Available

Characterization of the epoxy/silica hybrid nanocomposites; Thermal analysis; FTIR spectra of the uncured resin EPO_uncured (red), the cured resin EPO (yellow) and the in situ silica-epoxy system EPO6Si_1.25; WAXS pattern of epoxy-silica nanocomposite (measured by a 2-circle slit diffractometer [ThetaTheta]) and comparison of the results obtained by the Ganesha instrument; Determination of lattice plane distance from HRTEM images of the nanocomposite by using Image J software; Glass transition temperature (*T*_g_) of epoxy pristine resin and epoxy/silica nanocomposite; Storage modulus of epoxy pristine resin and epoxy/silica nanocomposite (PDF)
