# Supplementary material for: Structure and Bottom-up Formation Mechanism of Multisheet Silica-Based Nanoparticles Formed in an Epoxy Matrix through an In Situ Process
Source: Langmuir. 2021 Jul 18;37(29):8886–93. doi: 10.1021/acs.langmuir.1c01363 (PMC8397334; doi:10.1021/acs.langmuir.1c01363)
Supplement: Supplementary file 1 — la1c01363_si_001.pdf [file la1c01363_si_001.pdf]

Supporting Information

Structure and bottom-up formation mechanism of multi-sheet silica based nanoparticles formed in epoxy matrix through an in situ process

Francesco Branda[a],*, Aurelio Bifulco[a], Dieter Jehnichen[b], Dambarudhar Parida[c], Robin Pauer[d], Jessica Passaro[a], Sabyasachi Gaan[c], Doris Pospiech[e], Massimo Durante[a]

[a] Department of Chemical Materials and Industrial Production Engineering (DICMaPI) University of Naples Federico II, Naples, Italy
[b] Department Nanostructured Materials, Leibniz-Institut für Polymerforschung Dresden e. V., Hohe Str. 6, 01069 Dresden, Germany
[c] Laboratory for Advanced Fibers, Empa Swiss Federal Laboratories for Materials Science and Technology, Lerchenfeldstrasse 5, 9014 St., Gallen, Switzerland
[d] Advanced Materials and Surfaces, Empa, Swiss Federal Laboratories for Materials Science and Technology, Dubendorf, CH-8600, Switzerland.
[e] Department Polymer Structures, Leibniz-Institut für Polymerforschung Dresden e. V., Hohe Str. 6, 01069 Dresden, Germany
*Corresponding author

**Corresponding author*

branda@unina.it

Index

S1. Characterization of the epoxy/silica hybrid nanocomposites 1

Fig. S1. FTIR spectra of the uncured resin EPO_uncured (red), the cured resin EPO (yellow) and the in-situ silica-epoxy system EPO6Si_1.25 (blue)..1

Fig. S2. WAXS pattern of epoxy-silica nanocomposite (measured by a 2-circle slit diffractometer [ThetaTheta]) and comparison of the results obtained by the Ganesha instrument. 1

Fig. S3. Determination of lattice plane distance from HRTEM images of the nanocomposite by using Image J software. 2

S2. Thermal Analysis 3

Fig. S4. Glass transition temperature (Tg) of epoxy pristine resin and epoxy/silica nanocomposite. ..3

Fig. S5. Storage modulus of epoxy pristine resin and epoxy/silica nanocomposite..............................3

S1. Characterization of the epoxy/silica hybrid nanocomposites

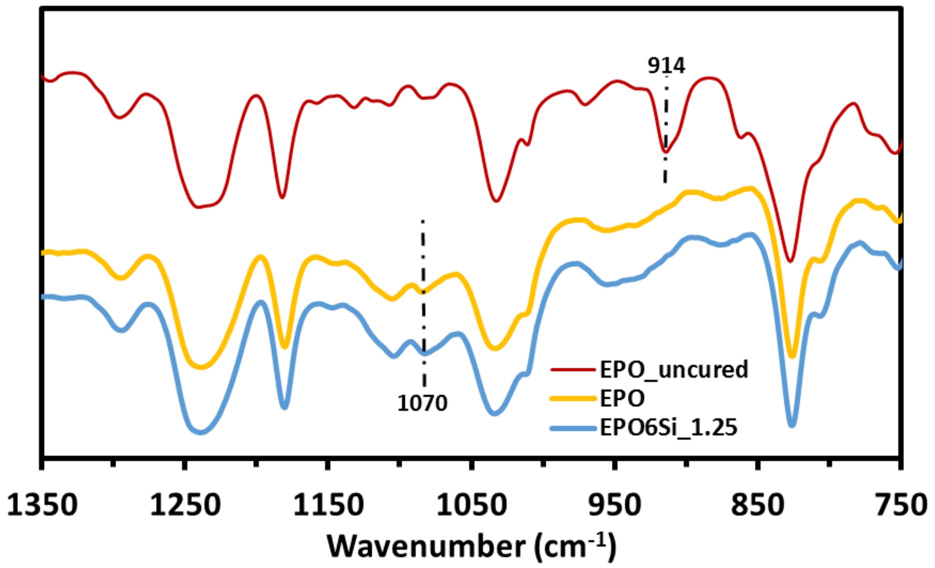

Fig. S1. FTIR spectra of the uncured resin EPO_uncured (red), the cured resin EPO (yellow) and the in-situ silica-epoxy system EPO6Si_1.25 (blue).

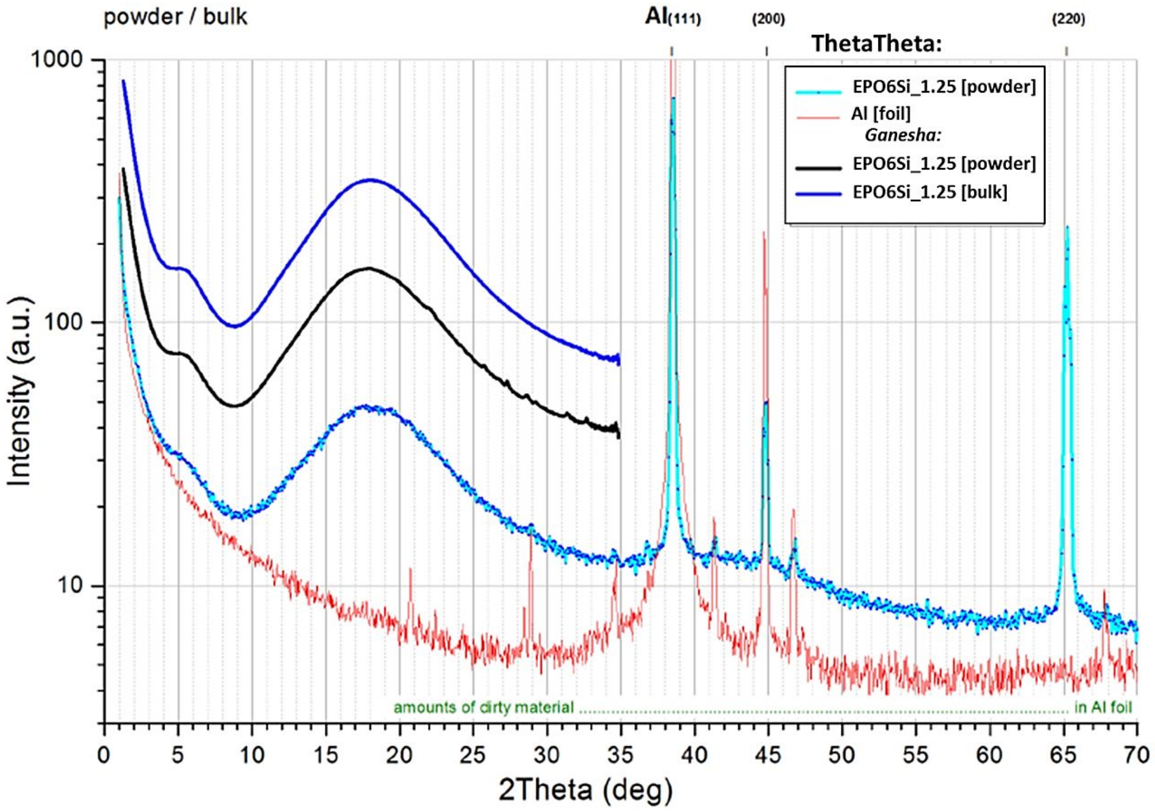

Fig. S2. WAXS pattern of epoxy-silica nanocomposite (measured by a 2-circle slit diffractometer [ThetaTheta]) and comparison of the results obtained by the Ganesha instrument.

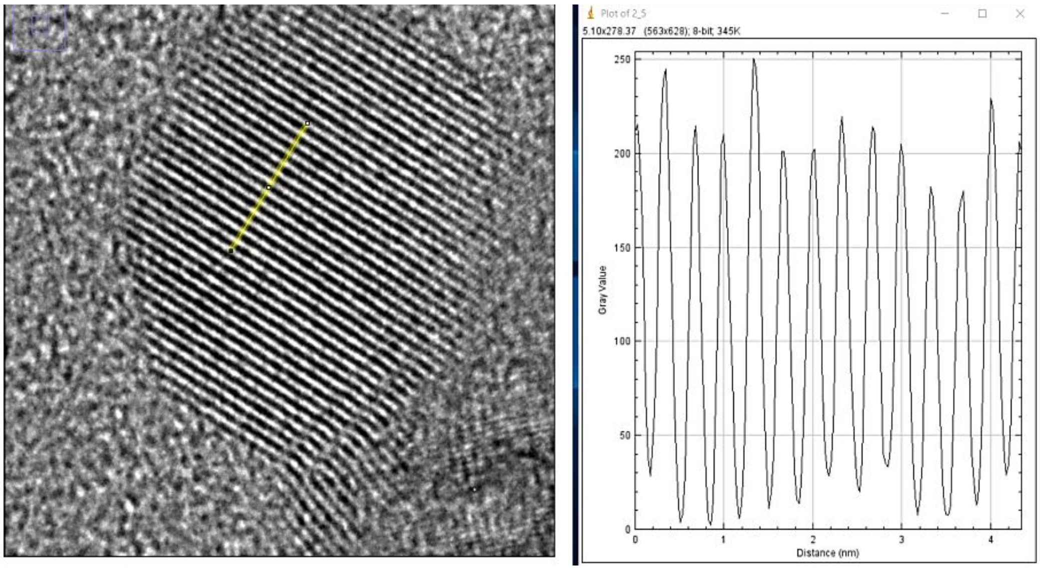

Fig. S3. Determination of lattice plane distance from HRTEM images of the nanocomposite by using Image J software.

S2. Thermal Analysis

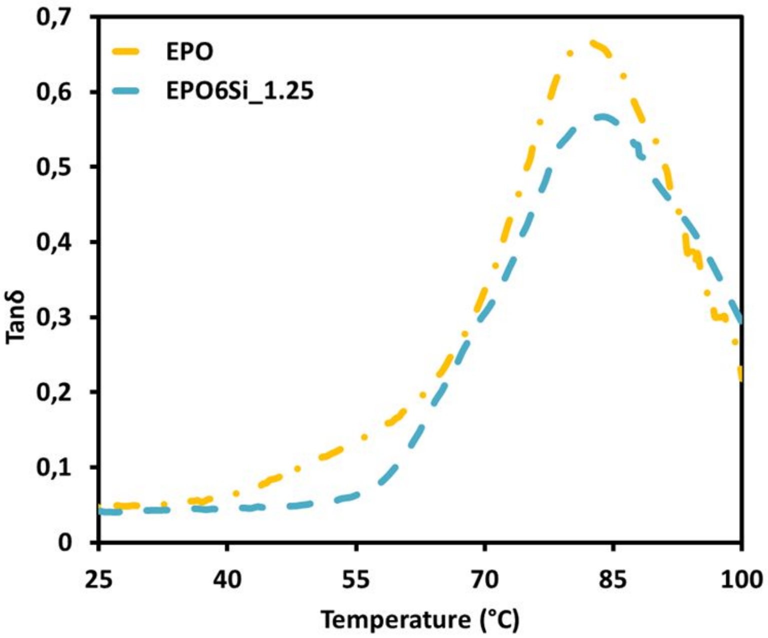

Fig. S4. Glass transition temperature (Tg) of epoxy pristine resin and epoxy/silica nanocomposite.

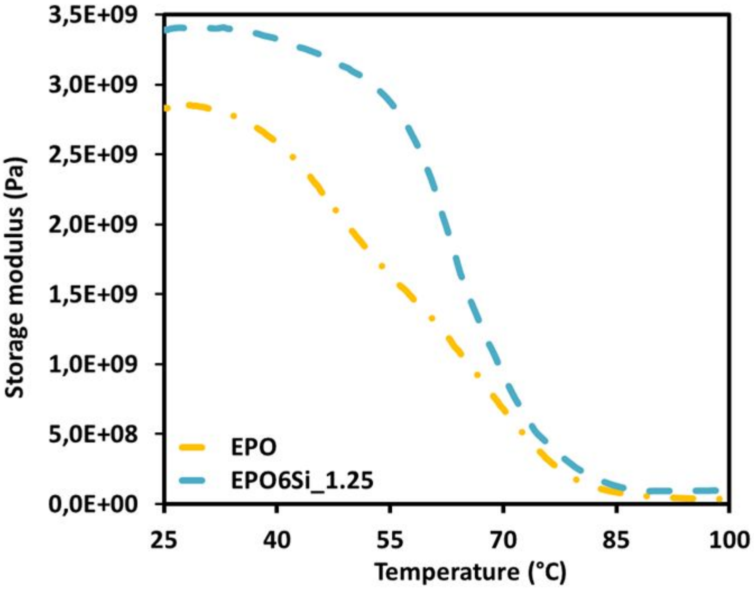

Fig. S5. Storage modulus of epoxy pristine resin and epoxy/silica nanocomposite.
